# Antioxidant Activities and Protective Effects of Dendropachol, a New Bisbibenzyl Compound from *Dendrobium pachyglossum*, on Hydrogen Peroxide-Induced Oxidative Stress in HaCaT Keratinocytes

**DOI:** 10.3390/antiox10020252

**Published:** 2021-02-06

**Authors:** Sakan Warinhomhoun, Chawanphat Muangnoi, Visarut Buranasudja, Wanwimon Mekboonsonglarp, Pornchai Rojsitthisak, Kittisak Likhitwitayawuid, Boonchoo Sritularak

**Affiliations:** 1 Department of Pharmacognosy and Pharmaceutical Botany, Faculty of Pharmaceutical Sciences, Chulalongkorn University, Bangkok 10330, Thailand; sakan.cu@gmail.com (S.W.); kittisak.l@chula.ac.th (K.L.); 2 Institute of Nutrition, Mahidol University, Nakhon Pathom 73170, Thailand; chawanphat.mua@mahidol.ac.th; 3 Natural Products for Ageing and Chronic Diseases Research Unit, Faculty of Pharmaceutical Sciences, Chulalongkorn University, Bangkok 10330, Thailand; pornchai.r@chula.ac.th; 4 Department of Pharmacology and Physiology, Faculty of Pharmaceutical Sciences, Chulalongkorn University, Bangkok 10330, Thailand; visarut.b@pharm.chula.ac.th; 5 Scientific and Technological Research Equipment Centre, Chulalongkorn University, Bangkok 10330, Thailand; wanwimon.m@chula.ac.th; 6 Department of Food and Pharmaceutical Chemistry, Faculty of Pharmaceutical Sciences, Chulalongkorn University, Bangkok 10330, Thailand

**Keywords:** *Dendrobium pachyglossum*, Orchidaceae, dendropachol, bisbibenzyl, antioxidant

## Abstract

Five compounds including a new bisbibenzyl named dendropachol (**1**) and four known compounds (**2–5**) comprising 4,5-dihydroxy-2,3-dimethoxy-9,10-dihydrophenanthrene (**2**), gigantol (**3**), moscatilin (**4**) and 4,5,4′-trihydroxy-3,3′-dimethoxybibenzyl (**5**) were isolated from a methanolic extract of *Dendrobium pachyglossum* (Orchidaceae). The chemical structures of the isolated compounds were characterized by spectroscopic methods. Dendropachol (**1**) was investigated for its protective effects on hydrogen peroxide (H_2_O_2_)-induced oxidative stress in HaCaT keratinocytes. Compound **1** showed strong free radical scavenging compared to the positive control. For the cytoprotective effect, compound **1** increased the activities of GPx and CAT and the level of GSH but reduced intracellular reactive oxygen species (ROS) generation and accumulation. In addition, compound **1** significantly diminished the expression of p53, Bax, and cytochrome C proteins, decreased the activities of caspase-3 and caspase-9, and increased Bcl-2 protein. The results suggested that compound **1** exhibited antioxidant activities and protective effects in keratinocytes against oxidative stress induced by H_2_O_2_.

## 1. Introduction

Skin is the largest organ of the human body. It covers the exterior surface of the body and functions as a preventive barrier against environmental insults. The skin consists of three main layers: epidermis, dermis, and hypodermis. The epidermis is the outermost layer of human skin and continuously exposed to external stimuli, such as harmful chemicals, microorganisms, and solar ultraviolet radiation [1]. Keratinocytes are the main cellular component of the epidermis. They function as the skin’s barrier to prevent damage caused by external factors. Under severe injury conditions, for example, continuous exposure to the harmful free radicals, keratinocytes may respond by self-healing or initiating apoptosis [2]. Disruption of keratinocytes can lead to impairment of the skin barrier and, eventually, skin aging [3].

Reactive oxygen species (ROS) play a crucial role in environmental stress. Normally, ROS are generated by mitochondria oxidative metabolisms in our body and various external stresses. At a low concentration, ROS are essential for retaining the normal physiological function responsible for cell development, including cell cycle progression, proliferation, differentiation, migration, and cell death [4]. ROS also provides defense mechanisms against microbes within the epithelial layer. However, excessive ROS can overcome antioxidant capacity, leading to cellular oxidative stress. The skin cells have evolved a variety of antioxidant enzymes to control ROS production and propagation. The antioxidant activities in the skin are mainly included in the numerously expressed antioxidant enzymes catalase (CAT), glutathione (GSH), and glutathione peroxidase (GPx), which responsible for balancing ROS by converting lipid hydroperoxide and H_2_O_2_ into water and/or oxygen [5,6,7]. The enhanced ROS accumulation leads to the oxidative stress in epidermal keratinocytes resulting in flattening of the dermal-epidermal junction, decreasing barrier function, and reducing the trans-epidermal water loss, as well as increasing pigmentation, which is implicated in aged skin [8,9]. Therefore, the balance between ROS production and antioxidant enzyme activity is an important strategy for the intervention of oxidative stress in HaCaT keratinocytes. 

Several types of phytochemicals from natural sources such as anthocyanins, phenolics, diterpenoids, and curcuminoids have been shown to protect keratinocytes against the oxidative stress caused by ROS [10,11,12,13]. *Dendrobium* is one of the largest and most important genera in the family Orchidaceae, with approximately 1400 species. More than 1100 species are mainly dispersed in Asia and Australia, of which about 150 species are widely distributed in Thailand [14,15]. The plants in this genus produce various types of compounds, including alkaloids, bibenzyls, fluorenones, phenanthrenes, sesquiterpenoids, polysaccharides, and amino acids [16]. Previous studies revealed that *Dendrobium* plants are a good source for skin anti-aging agents. The crude extracts from *D.* *sonia* “earsakul” (a *Dendrobium* hybrid) and the bioactive constituents of *D. loddigesii* have been found to inhibit matrix metalloproteinase enzymes (MMP) and stimulate the production of collagen in human dermal fibroblasts [17,18]. Crude extracts of *D. tosaense, D. loddigesii,* and *D.* *sonia* “earsakul” showed inhibitory effects on melanogenesis [17,18,19]. Polysaccharides isolated from *D. denneanum, D. officinale,* and the crude extracts of *D.* *sabin* (a *Dendrobium* hybrid) and *D. moniliforme* showed *in vitro* antioxidant activities [20,21,22,23]. However, the protective effects of compounds from *Dendrobium* plants against the ROS-induced oxidative stress in HaCaT cells have not been reported.

*Dendrobium pachyglossum* Par. & Rchb.f., known in Thai as Ueang Khon Mu, is distributed in northeastern, eastern, and south of Thailand [24]. Up to the present, there have been no reports on the chemical constituents and biological activities of this plant. In the present study, the MeOH extract prepared from whole plants of *D. pachyglossum* was screened for cytoprotective effects against hydrogen peroxide (H_2_O_2_) HaCaT keratinocytes and found to improve the percentage of cell viability as compared with the H_2_O_2_-treated group. Chromatographic separation of this MeOH extract led to the isolation of a new bisbibenzyl compound named dendropachol (**1**), along with four known compounds (**2–5**). The underlying mechanisms of antioxidant activities and protective effects of compound **1** in HaCaT cells against oxidative stress induced by hydrogen peroxide were determined.

## 2. Materials and Methods

### 2.1. General Experimental Procedures

UV spectra were recorded on a Milton Roy Spectronic 300 Array spectrophotometer (Rochester, Monroe, NY, USA), and IR spectra were obtained on a Perkin-Elmer FT-IR 1760X spectrophotometer (Norwalk, Fairfield, CT, USA). Optical rotation was measured on a Perkin-Elmer 341 polarimeter (Norwalk, Fairfield, CT, USA). Mass spectra were recorded on a Bruker micro TOF mass spectrometer (ESI-QqTOF-MS) (Manchester, UK). NMR spectra were recorded on a Bruker Avance DPX-300 FT-NMR spectrometer or a Bruker Avance III HD 500 NMR spectrometer (Rheinstetten, Germany). Column chromatography (CC) was performed on silica gel 60 (Kieselgel 60, 70–320 µm, Merck, Darmstadt, Germany), silica gel 60 (Kieselgel 60, 230–400 µm, Merck, Darmstadt, Germany), C-18 (Kieselgel 60 RP-18, 40–63 µm, Merck, Darmstadt, Germany), Diaion HP (Mitsubishi Chemical, Tokyo, Japan) and Sephadex LH-20 (25–100 µm, GE Healthcare, Göteborg, Sweden).

### 2.2. Plant Materials

The whole plant of *D. pachyglossum* was purchased from Chatuchak market, Bangkok, in July 2015. Plant identification was done by B. Sritularak. A voucher specimen (BS-DPachy-072558) has been deposited at the Department of Pharmacognosy and Pharmaceutical Botany, Faculty of Pharmaceutical Sciences, Chulalongkorn University.

### 2.3. Extraction and Isolation

The dried powdered whole plant of *Dendrobium pachyglossum* (2.7 kg) was extracted with MeOH at room temperature, yielding a MeOH extract (220 g). The MeOH extract was suspended in water and partitioned with EtOAc and *n*-BuOH to give an EtOAc extract (110 g), an *n*-BuOH extract (35 g), and an aqueous extract (55 g). The EtOAc extract was initially subjected to vacuum liquid chromatography on silica gel (EtOAc-hexane, gradient) to give 6 fractions (A–F). Fraction C (17.7 g) was fractionated by column chromatography (CC) over silica gel (EtOAc-hexane, gradient) and then purified by Sephadex LH-20 (acetone) to give 4,5-dihydroxy-2,3-dimethoxy-9,10-dihydrophenanthrene (**2**) (7 mg). Fraction D (4.0 g) was separated by Sephadex LH-20 (MeOH) and then purified again by Sephadex LH-20 (MeOH) to yield gigantol (**3**) (57 mg) and moscatilin (**4**) (46 mg). Separation of fraction E (11 g) was performed by CC over silica gel, eluted with MeOH-CH_2_Cl_2_ (gradient) to give 4 fractions (E1-E4). Fraction E2 (980 mg) was further separated on Sephadex LH-20 (acetone) and then by CC (silica gel, MeOH-CH_2_Cl_2_, gradient) to yield 4,5,4′-trihydroxy-3,3′-dimethoxybibenzyl (**5**) (263 mg). Compound **1** (9 mg) was obtained from fraction E5 (527 mg) after separation by CC (silica gel, acetone-hexane) and Sephadex LH-20 (MeOH).

Dendropachol (**1**) Brown amorphous solid; ${\left[\alpha \right]}_{D}^{20}$ + 4.4 (c 0.01, MeOH); UV (MeOH) λ_max_ (log ε): 204 (4.10), 281 (3.28) nm; IR (film) λ_max_: 3355, 2923, 2852, 1709, 1606, 1512, 1450, 1431, 1268, 1209, 1124 cm^−1^; ESI-QqTOF-MS: [M-H]^−^ at *m/z* 575.1906 (calculated for C_32_H_31_O_10_, 575.1917); ^1^H NMR (500 MHz, acetone-*d*_6_) and ^13^C NMR (125 MHz, acetone-*d*_6_) spectral data, see (Table 1).

### 2.4. Cell Culture

The immortalized human epidermal keratinocyte (HaCaT) cell line was obtained from Thermo Fisher Scientific (Waltham, MA, USA). The HaCaT cells were cultured in complete media, which comprised of Dulbecco’s modified Eagle’s medium (DMEM) supplemented with 10% (*v/v*) heat-inactivated fetal bovine serum and 1% (*v/v*) penicillin-streptomycin at 37 °C in a humidified atmosphere of 5% CO_2_/95% air. All reagents were purchased from Invitrogen (Grand Island, NY, USA). 

### 2.5. Cytotoxic Effect of Chemical Constituents from D. pachyglossum on HaCaT Keratinocytes Cells

The cytotoxic effect of the isolated compounds **1–5** on cell viability of HaCaT cells was evaluated using the 3-(4,5-dimethlthiazol-2-yl)-2,5-diphenyl tetrazolium bromide tetrazolium (MTT) assay. The HaCaT cells were seeded in 96-well plates at a density of 3.0 × 10^4^ cells/well and incubated at 37 °C in a humidified atmosphere of 5% CO_2_ for 24 h. After seeding for 24 h, the media was removed, and the cells were washed with serum-free media. Subsequently, the cells were incubated with compounds **1–5** at the concentrations of 50 and 100 µg/mL in serum-free media for 24 h. The 0.5% DMSO was used as a control. After incubation, the cells were washed and incubated in serum-free media containing 0.5 mg/mL of an MTT solution at 37 °C in a humidified atmosphere of 5% CO_2_/95% air for 4 h. Subsequently, the media was removed, and 200 µL of DMSO was added to each well to dissolve the formazan crystals. The absorbance of formazan was measured at 540 nm using a microplate reader (CLARIOstar, BMG Labtech, Ortenberg, Germany). Four replicates of each experiment were performed. 

### 2.6. Effect of H_2_O_2_ on Cell Viability of HaCaT Keratinocyte Cells

To determine the concentration of H_2_O_2_ required to reduce the cell viability of HaCaT cells by 50%, the cells were treated with different concentrations of H_2_O_2_ (100, 200, 300, 400, and 500 µmol/L) in serum-free media at 37 °C for 1 h. The serum-free medium without H_2_O_2_ was used as a control. After incubation, the cells were washed twice with an excess of PBS, and the cell viability was measured using the MTT assay.

### 2.7. Effect of MeOH Extract and Compound ***1*** on Cell Viability of HaCaT Cells under Oxidative Stress

The HaCaT cells were seeded in 96-well plates at a cell density of 3.0 × 10^4^ cells/well. The cells were treated with serum-free media containing MeOH extract (500 µg/mL) and compound **1** (12.5, 25 and 50 µg/mL) for 24 h and then washed with PBS. Subsequently, the cells were added with H_2_O_2_ at the concentration of 500 µmol/L in serum-free media prior to incubation at 37 °C for 1 h. The cell viability was determined using the MTT assay measured at 540 nm. DMSO (0.5% *v/v*) was used as a control.

### 2.8. Antioxidant Enzyme Activities 

The HaCaT cells were seeded at 1.0 × 10^6^ cells/well in 6-well plates for 24 h. After washing with serum-free media, the cells were treated with compound **1** at the concentrations of 12.5, 25, and 50 µg/mL for 24 h. Then, H_2_O_2_ (500 µmol/L in serum-free media) was added, and the cells were incubated at 37 °C for 1 h. After incubation, the cells were resuspended in an ice-cold lysis buffer at 37 °C for 5 min and centrifugated at 13,500× *g* at 4 °C for 5 min to obtain cell lysate for determination of antioxidant enzyme activities and GSH level. The activities of glutathione peroxidase (GPx) and catalase (CAT) and the level of glutathione (GSH) were measured using GPx, GSH, and CAT cellular activity assay kits (Cayman Chemical, Ann Arbor, MI, USA).

### 2.9. Effect of Compound ***1*** on DPPH (2,2-Diphenyl-1-Picrylhydrazyl) Free Radical Scavenging Activity

The free radical scavenging activity of compound **1** was examined by DPPH assay with some modifications. Briefly, the compound **1** solution at the concentrations range of 5–15 µg/mL was added to 6.0 × 10^−5^ mol/L of DPPH solution in a 96-well plate. The mixture was incubated for 30 min at room temperature and protected from light. When DPPH receives a hydrogen atom from an antioxidant sample, its color changes from violet to light yellow. The absorbance was measured at 517 nm with a microplate reader. Ascorbic acid at a concentration between 3.5–17.5 µg/mL was used as a positive control. The percentage of scavenging activity (%SA) was calculated using the following equation. The IC_50_ value of each sample was determined from the graph plotted between concentration and the percentage of inhibition.

%SA = [(A_sample_ − A_blank_)/A_blank_] × 100

where A_sample_ is the absorbance of DPPH treated with the sample at 517 nm, A_blank_ is the absorbance of DPPH treated with methanol at 517 nm.

### 2.10. Effect of Compound ***1*** on ROS Production in HaCaT Cells under Oxidative Stress

Intracellular ROS concentrations were assessed using a dichloro-dihydro-fluorescein diacetate (DCFH-DA) assay. The HaCaT cells were seeded at 3.0 × 10^4^ cells/well in black 96-well plates for 24 h. After incubation, the medium was removed. The cells were washed with an excess of PBS and treated with compound **1** in serum-free media at the concentrations of 12.5, 25, and 50 µg/mL for 24 h. DMSO (0.5% *v/v*) was used as a control. The cells were then washed with an excess of PBS, pre-treated with 10 µmol/L of DCFH-DA in serum-free media at 37 °C for 20 min. After washing with PBS, the cells were added with H_2_O_2_ at the concentration of 500 µmol/L in serum-free medium at 37 °C for 1 h. Finally, the cells were washed twice with PBS, and the ROS levels were measured using the microplate reader at the excitation and emission wavelength of 485 and 530 nm, respectively.

### 2.11. Anticaspase-3 and -9 Activities

To evaluate the effects of compound **1** on anticaspase-3 and-9 activities, the HaCaT cells were seeded in 6-well plates with 1.0 × 10^6^ cells/well for 24 h. After incubation, the cells were treated with compound **1** at the concentrations of 12.5, 25, and 50 µg/mL for 24 h. The treated cells were homogenized in a hypotonic buffer (20 mM Tris-HCl pH 7.5, 1 mM ethylenediaminetetraacetic acid, 100 µmol/L phenylmethanesulfonylfluoride, 2 µg/mL aprotinin, pepstatin, and leupeptin) to obtain the supernatant part. The supernatant was added with a specific substrate (*N*-acetyl-Asp-Glu-Val-Asp *p*-nitroanilide or *N*-acetyl-Leu-Glu-His-Asp *p*-nitroanilide for caspase-3 or caspase-9, respectively) at the concentration of 100 µmol/L. The mixture was then incubated at 37 °C for 1 h prior to absorbance measurement at 450 nm using the microplate reader. 

### 2.12. Protein Extraction and Western Blot Analysis

The HaCaT cells were lysed in a radioimmunoprecipitation (RIPA) buffer for 30 min on ice, followed by centrifugation at 13,500× *g* at 4 °C for 5 min. The whole-cell lysates containing 40 µg of protein were separated by SDS-PAGE (10% gel) and transferred onto a nitrocellulose membrane. The membranes were then blocked with 5% nonfat dry milk in PBS and incubated overnight with primary antibodies against cleaved Bax (1:1000), BCL-2 (1:1000), cytochrome C (1:1000), p53 (1:1000), and β-actin (1:20,000) at 4 °C. After washing with TBST, the membranes were incubated for 2 min with the species-specific horseradish peroxide conjugated secondary antibody reacting with SuperSignal solution (Endogen Inc, Rockford, IL, USA). Then, the membranes were exposed to X-ray film. The membranes were then stripped off the bond antibodies and re-probed with anti-β actin antibody to ensure the equal loading of the protein. The densities of target bands were quantified by the Image J program (freeware downloads from http://rsb.info.nih.gov/ij/ (accessed on 25 December 2020)). The results were expressed as a relative band intensity ratio of the target proteins against that of β-actin.

### 2.13. Statistical Analysis

All of the data were performed at least in three replicates. Comparisons between groups were performed using the GraphPad Prism software Version 8.00 for Mac (GraphPad Software, Inc., San Diego, CA, USA). Values were presented as mean ± standard deviation (SD). Means were compared by one-way analysis of variance (ANOVA) with Dunnett’s test, and differences were considered significant at *p* < 0.05. 

## 3. Results

### 3.1. Structural Characterization

**Figure 1:**
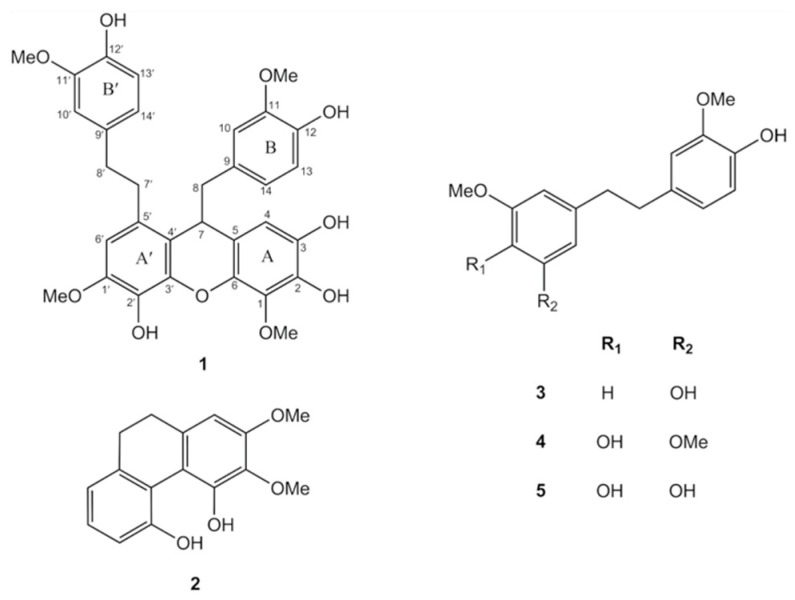
Structures of compounds **1–5** isolated from the EtOAc extract of *D. pachyglossum*.

The MeOH extract prepared from the whole plant of *D. pachyglossum* was found to protective effects against H_2_O_2_ on induced oxidative stress in HaCaT keratinocytes. The percentage of cell viability significantly increased to 70.13 ± 1.25% at 500 µg/mL compared to the H_2_O_2_-induced oxidative stress in HaCaT keratinocytes (50.81 ± 1.12%). Therefore, the extracts were then sequentially partitioned with various solvents to give EtOAc, *n*-butanol, and aqueous extracts. The phytochemical investigation of the EtOAc extract resulted in the isolation of a new bisbibenzyl compound (**1**), along with four known compounds, including 4,5-dihydroxy-2,3-dimethoxy- 9,10-dihydrophenanthrene (**2**) [25], gigantol (**3**) [26], moscatilin (**4**) [27], and 4,5,4′-trihydroxy-3,3′-dimethoxybibenzyl (**5**) [28]. Their structures were elucidated through analysis of their spectroscopic data (Figure 1).

Compound **1** was obtained as a brown amorphous solid. The negative ESI-QqTOF-MS showed an [M-H]^–^ at *m/z* 575.1906 (calculated for C_32_H_31_O_10_, 575.1917). The IR spectrum exhibited absorption bands for hydroxyl (3355 cm^−1^), aromatic ring (2923, 1606 cm^−1^), methylene (1450 cm^−1^) and ether (1268 cm^−1^) functionalities. The UV absorptions at 204 and 281 nm suggested a bisbibenzyl nucleus of the compound **1** [29]. The ^1^H NMR (Table 1) confirmed the existence of a bisbibenzyl skeleton by the presence of signals of aliphatic protons at δ 2.67, 2.74 (2H, m, H-8), 2.76, 2.84 (2H, m, H-7′), 2.80 (2H, m, H-8′) and 4.12 (1H, dd, *J* = 7.0, 5.5 Hz, H-7) with HSQC correlations to carbon atoms at δ 45.9 (C-8), 34.5 (C-7′), 38.0 (C-8′) and 39.7 (C-7), respectively [30]. The ^1^H NMR spectrum (Table 1) also showed eight aromatic proton signals at δ 6.13–6.77 and resonances for four methoxyl groups at δ 3.56 (3H, s, MeO-11), 3.76 (3H, s, MeO-11′), 3.81 (3H, s, MeO-1′) and 3.89 (3H, s, MeO-1). Compared with the ^1^H and ^13^C NMR spectra of dendrofalconerol A, a bisbibenzyl derivative isolated from *D. falconeri* revealed the structural similarity with compound **1**, particularly in rings A and A′ based on the substitution patterns and the points of connection [30]. Compound **1** had rings A connect to ring A′ through a methane bridge and an ether linkage, as shown by the HMBC correlations from H-7 to C-4 (δ 109.7), C-6 (δ 140.0), C-9 (δ 130.9), C-3′ (δ 142.4) and C-5′ (δ 129.8) (Table 1). On the ring A of compound **1**, H-4 (1H, δ 6.21, s) exhibited a NOESY correlation with H-7, and HMBC correlations with C-2 (δ 136.9), C-6 (δ 140.0) and C-7 (δ 39.7). The NMR signal of MeO-1 protons appeared at δ 3.89 (3H, s). For the ring A′, the ^1^H NMR signal at δ 6.66 (1H, s) was assigned to H-6′ based on its 3-bond couplings to C-2′ (δ 134.0), C-4′ (δ 119.0) and C-7′ (δ 34.5). The presence of a methoxyl at C-1′ (δ 3.89) was confirmed by its NOESY cross-peak with H-6′. For the ring B, ^1^H NMR showed signals for two doublets at δ 6.13 (1H, *J* = 2.0 Hz, H-10) and δ 6.56 (1H, *J* = 8.0 Hz, H-13), a double doublet at δ 6.22 (1H, *J* = 8.0, 2.0 Hz, H-14) and a methoxy protons at δ 3.56 (3H, s, MeO-11). The HMBC correlations of H-10 and H-14 with C-8 indicated that the ring B was di-oxygenated with a hydroxyl group or a methoxyl group at C-11 and C-12. A NOESY cross-peak of the methoxyl group to H-10, suggesting the methoxyl group at C-11. The ^1^H NMR ABM spin system also appeared for the ring B′ at δ 6.69 (1H, dd, *J* = 8.5, 2.0 Hz, H-14′), 6.71 (1H, d, *J* = 8.5 Hz, H-13′) and 6.77 (1H, d, *J* = 2.0 Hz, H-10′). The HMBC correlations of C-8′ (δ 38.0) with H-10′ and H-14′ confirmed that the ring B′ was di-oxygenated substitution similar to ring the B. The fourth methoxyl group was located on the ring B′ at C-11′ based on its NOESY correlation with H-10′. Based on the above spectral evidence, the structure of compound **1** was established, as shown in Figure 1, and the compound was given the trivial name dendropachol. 

### 3.2. Cytotoxicity of Isolated Compounds ***1*** from D. pachyglossum on HaCaT Keratinocytes

**Figure 2:**
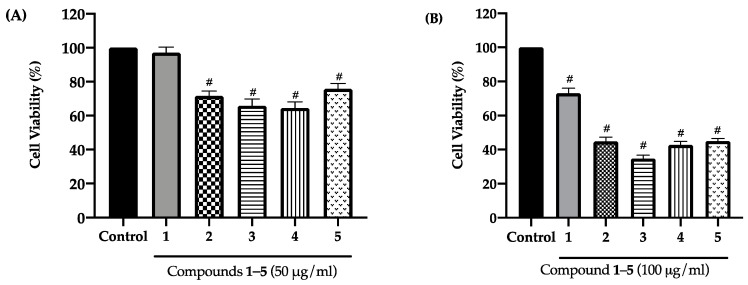
Effect of compounds **1–5** on cell viability of HaCaT keratinocytes. Cells were treated with a concentration (**A**) 50 and (**B**) 100 µg/mL of the test sample for 24 h. After treatment, the percent cell viability was measured using the 3-(4,5-dimethlthiazol-2-yl)-2,5-diphenyl tetrazolium bromide tetrazolium (MTT) assay. Graphs showed mean ± SD values of four replication. *#* *p* < 0.05 indicates significant differences from the untreated group.

The isolated compounds **1–5** were studied for cytotoxicity on HaCaT keratinocytes using the MTT-assay. Each compound was initially evaluated at concentrations of 50 and 100 µg/mL. When tested at 50 µg /mL, only compound **1** did not exhibit cytotoxicity (*p* > 0.05), showing 97.16 ± 3.19 percent of cell viability; meanwhile, compound **2–5** significantly differences (*p* < 0.05) reducing cell viability between 65 ± 1.80% to 75 ± 1.59% as compared to the control (Figure 2A). After pre-treatment with all compounds (**1–5**) at maximum concentration 100 µg/mL, the viabilities of HaCaT keratinocytes significantly (*p* > 0.05) reduced lower than 80% (73.13 ± 1.44% to 34.70 ± 1.08%) compared to the untreated group (Figure 2B). Therefore compound **1** at concentration 50 µg/mL was selected for further study.

### 3.3. DPPH Radical Scavenging Activity of Compound ***1***

DPPH assay is based on the capacity of the sample to scavenge DPPH radical. Compound **1** was evaluated for this activity compared to ascorbic acid as the positive control. Compound **1** at a concentration range of 5–15 µg/mL exhibited %SA from 19.21 ± 0.13% to 78.96 ± 0.81%, while 3.5–17.5 µg/mL of ascorbic acid produced %SA from 15.69 ± 0.21% to 82.01 ± 0.71%. The IC_50_ value of compound **1** was 11.13 ± 0.32 µg/mL, which is comparable to that of ascorbic acid (IC_50_ value 10.77 ± 0.64 µg/mL). These results suggest that compound **1** is a strong radical scavenger.

### 3.4. Effects of Compound ***1*** on Cell Viability of HaCaT Keratinocytes and H_2_O_2_-Induced Oxidative Stress

**Figure 3:**
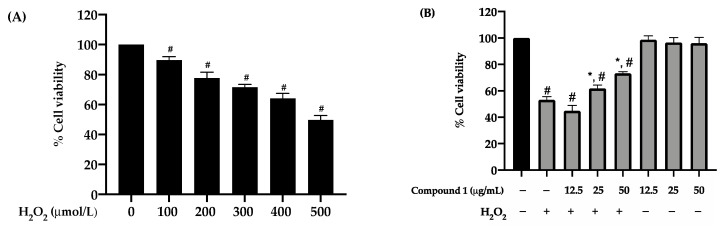
Effect of compound **1** on cell viability in HaCaT keratinocytes induced oxidative stress with H_2_O_2_. (**A**) Cells were treated with H_2_O_2_ (100–500 µmol/L) for 1 h. (**B**) Cells were pre-treated with compound **1** (12.5, 25, and 50 µg/mL) prior to exposure to 500 µmol/L H_2_O_2_ for 24 h. After the treatment, the percent cell viability was measured using the MTT assay. Graphs exhibited mean ± SD values of four replications. * p < 0.05 indicates significant differences from the H_2_O_2_ induction group, *# p* < 0.05 indicates significant differences from the control group.

HaCaT keratinocytes were treated with different concentrations of H_2_O_2_ (100, 200, 300, 400, and 500 µmol/L) as described in Section 2.6. The results showed that 500 µmol/L of H_2_O_2_ decreased cell viability to ~50% compared to the untreated group (Figure 3A), and therefore, this concentration was used for further studies. Furthermore, we found that compound **1** at 25 and 50 µg/mL significantly (*p* < 0.05) increased cell viability to 61.83 ± 2.51% and 73.22 ± 1.30%, respectively (Figure 3B). The results suggest that compound **1** protects HaCaT keratinocyte cells by preventing H_2_O-induced oxidative stress. 

### 3.5. Effects of Compound ***1*** on GPx and CAT Activities, GSH Level, and ROS Production in H_2_O_2_-Treated HaCaT Keratinocytes

**Figure 4:**
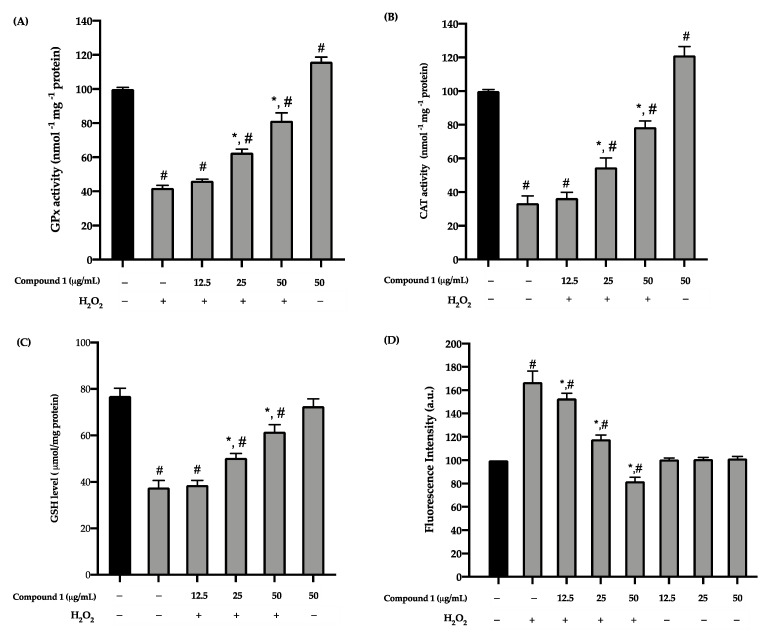
Effect of compound **1** on activities of antioxidant enzymes (**A**) GPx, (**B**) CAT, (**C**), GSH level, and (**D**) Intracellular ROS (fluorescence intensity) in HaCaT keratinocytes induced oxidative stress with H_2_O_2_. Cells were pre-treated with various concentrations of compound **1** (12.5, 25, and 50 µg/mL) for 24 h exposure to 500 µmol/L of H_2_O_2_ for 1 h. Graphs represent the mean ± SD values of four replications. * p < 0.05 indicates significant differences from the H_2_O_2_ stimulation group, *# p* < 0.05 indicates significant differences from the control group.

The effects of compound **1** on the activities of GPx and CAT and the level of GSH were determined. The exposure of HaCaT keratinocytes to H_2_O_2_-induced oxidative stress significantly (*p* < 0.05) decreased the activities of GPx and CAT and the level of GSH compared to the control group. After pre-treatment with compound **1** at 25 and 50 µg/mL, the activities of GPx and CAT and the level of GSH were significantly increased (Figure 4A–C). 

To confirm the effects of compound **1** on the intracellular levels of ROS, HaCaT keratinocytes treated with compound **1** were induced with H_2_O_2_ for 24 h. Afterwards, the cells were stained with a DCF-DA solution, and the ROS levels were analyzed. In the untreated cells, H_2_O_2_ at a concentration of 500 µmol/L significantly increased the intracellular ROS levels in the HaCaT keratinocytes. Cells treated with compound **1** showed inhibitory effects on H_2_O_2_-induced ROS production in a dose-dependent manner (Figure 4D), consistent with the results acquired from the antioxidant enzyme assays. The evidence suggests that compound **1** protects HaCaT keratinocytes by scavenging the ROS produced in response to H_2_O_2_ exposure.

### 3.6. Effects of Compound ***1*** on the Expression of p53 Protein Induced by H_2_O_2_ in HaCaT Keratinocytes

**Figure 5:**
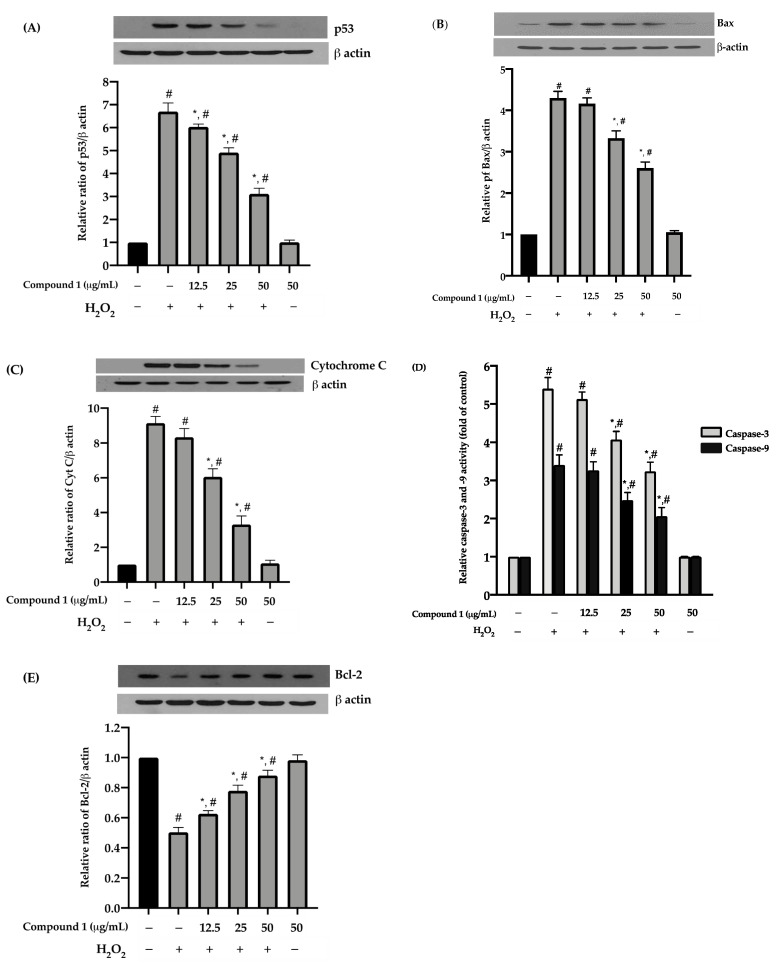
Effect of compound **1** on the expression of (**A**) p53, (**B**) Bax, (**C**) cytochrome C, (**D**) caspase-3 and -9, and (**E**) Bcl-2 in HaCaT keratinocytes induced oxidative stress with H_2_O_2_. Cells were pre-treated with various concentrations of compound **1** (12.5, 25, and 50 µg/mL) for 24 h exposed to 500 µmol/L of H_2_O_2_ for 1h. Graphs represent the mean ± SD values of four replications. * p < 0.05 indicates significant differences from the H_2_O_2_ stimulation group, *# p* < 0.05 indicates significant differences from the control group.

The effect of compound **1** on p53 in H_2_O_2_-treated HaCaT keratinocytes was determined by western blot analysis. As shown in Figure 5A, the expression level of p53 protein in H_2_O_2_-induced cells was significantly (*p* < 0.05) increased by 6.77-fold, compared to the untreated cells. However, the compound **1** pre-treatment significantly (*p* < 0.05) reduced the H_2_O_2_-induced p53 on HaCaT keratinocytes in a dose-dependent manner by 6.03, 4.92, and 3.12-folds, respectively (Figure 5A). The results indicated that compound **1** exhibited prevent HaCaT apoptosis through a decrease of the p53 expression.

### 3.7. Effects of Compound ***1*** on the Expression of Bax and Bcl-2 Proteins Induced by H_2_O_2_ in HaCaT Keratinocytes

To investigate whether apoptosis protection of compound **1** correlated to the regulation of the pro-apoptotic and anti-apoptotic proteins, we examined the expression of the Bax and Bcl-2 proteins expression in H_2_O_2_-induced HaCaT keratinocytes using western blot analysis. The cells exposed to H_2_O_2_ augmented the Bax and inhibited the expression of Bcl-2 protein by 4.3 and 0.5 -folds compared to the control, respectively, resulting in an imbalance between Bax and Bcl-2. Compound **1** pre-treatment at 25 and 50 µg/mL significantly (*p* < 0.05) decreased protein Bax by 3.23 and 2.60 -folds, while Bcl-2 protein significantly (*p* < 0.05) increased in a dose-dependent manner by 0.62, 0.78, and 0.88 -folds for compound **1** at 12.5, 25, and 50 µg/mL, respectively (Figure 5B,E). The results indicate that compound **1** exerted its protective effects on the H_2_O_2_-induced cells via modulation of Bax and Bcl-2, most likely at the transcriptional level.

### 3.8. Effects of Compound ***1*** on the Expression of Cytochrome C Protein Induced by H_2_O_2_ in HaCaT Keratinocytes

The association between the anti-apoptosis activity of compound **1** in H_2_O_2_-induced HaCaT keratinocytes and cytochrome C release was also investigated. Exposure of HaCaT cells to H_2_O_2_ significantly enhanced cytochrome C level by 9.14-fold compared to the control group (*p* < 0.05). Pre-treatment of HaCaT cells with compound **1** at 25 and 50 µg/mL significantly decrease cytochrome C level by 6.06 and 3.31 -folds (*p* < 0.05), respectively (Figure 5C). The results suggest that compound **1** could reduce cytochrome C protein in the H_2_O_2_-treated HaCaT keratinocytes.

### 3.9. Effects of Compound ***1*** on Caspase-3 and -9 Activities in H_2_O_2_-Induced HaCaT Keratinocytes

To investigate whether the anti-apoptotic property of compound **1** in H_2_O_2_-induced HaCaT keratinocytes was related to caspase-3 and caspase-9, we analyzed the activities of the two enzymes, and the results were shown in Figure 5D. The cells exposed to H_2_O_2_ increased the activities of caspase-3 and -9 by approximately 5.68 and 3.62-folds, respectively. Pre-treatment of the cells with compound **1** at 25 and 50 µg/mL significantly (*p* < 0.05) reduced the levels of caspase-3 and -9 activities. The results suggest that compound **1** inhibits H_2_O_2_-induced apoptosis in HaCaT keratinocytes by suppressing caspase-3 and -9 activities.

## 4. Discussion

*Dendrobium* spp. have been reported to possess many secondary metabolites with potent antioxidant activities [19,25], and thus, they appear to be a good source of natural skin rejuvenating and anti-aging agents. In the present study, we investigated the chemical constituents of *D. pachyglossum* and the biological activities of the isolated compounds, including the new bisbibenzyl named dendropachol (**1**), together with 4 known compounds (**2–5**). The imbalance in the level of oxidants and antioxidants in the cells is due to excessive production of ROS. In this study, H_2_O_2_ was chosen to induce oxidative stress in HaCaT keratinocytes because it is mainly accumulated in the epidermis layer of the skin and can diffuse freely in and out of the cells and tissues [31,32]. H_2_O_2_ is one of the most common oxidants used in the oxidative stress models [33]. The increase of the intracellular H_2_O_2_ level in response to various pro-oxidants can further induce excessive ROS production in the cells [31]. H_2_O_2_ and its corresponding ROS create oxidative stress in keratinocytes and lead to lipid peroxidation, cell integrity damage, and apoptosis induction, leading to skin aging [34]. To evaluate the suitable concentration of H_2_O_2_ to act as oxidative stress, based on to cause an ~50% reduction in HaCaT cell viability [35]. The concentration of H_2_O_2_ between 100–500 μmol/L is mainly used for inducing oxidative stress in cells [36]. The H_2_O_2_ concentration ranging from 100–500 μmol/L were evaluated to determine an appropriate concentration for inducing oxidative stress in HaCaT cells. The result showed that the viabilities of HaCaT cells decreased in a dose-dependent manner, and the highest concentration (500 µmol/L) caused about 50% of cell survival (Figure 3A). Our results agreed with previous studies showing that H_2_O_2_ at 500 μmol/L reduced cell viability in the range between 50–65% compared to the untreated group [37,38,39]. In the present study, the cytotoxicity of compounds **1–5** was evaluated on HaCaT keratinocytes before the preventive effect mechanism study. To confirm the non-toxicity of compounds **1–5**, we chose to use high concentrations (50 and 100 µg/mL) to ensure that they are safe for cosmetic applications even at high concentrations. Among five compounds, we found that only compound **1** (50 µg/mL) did not cause toxicity to HaCaT cells, while treatments with other compounds resulted in a significant reduction in viability (Figure 2A). The observed toxicity of compounds **2–5** was parallel to previous studies with other cell lines, including cancer cells [25,40,41,42]. The cytotoxicity of compounds **2–5** might be potentiated in H_2_O_2_-induced oxidative stress conditions. Therefore, our current study mainly focused on compound **1** at 50 µg/mL to further investigate its molecular mechanism to prevent H_2_O_2_-induced oxidative stress in HaCaT keratinocytes.

The DPPH assay was conducted to investigate the antioxidant activity of compound **1**. This assay is widely used to determine the antioxidant property of compounds as a free radical scavenger or hydrogen donors. The degree of DPPH decolorization is related to the scavenging potential of the tested compounds [43]. Compound **1** (IC_50_ value 11.13 ± 0.32 µg/mL) exhibited a strong DPPH radical scavenging effect compared to ascorbic acid (IC_50_ value 10.77 ± 0.64 µg/mL), indicating that compound **1** had strong antioxidant activity against DPPH. The antioxidant property of compound **1** was further investigated whether it could reduce the accumulated intracellular ROS in H_2_O_2_-induced HaCaT cells using the DCFH-DA assay. The pre-treatment of HaCaT cells with compound **1** effectively reduced cellular ROS production (Figure 3B and Figure 4D). The strong antioxidant activity of compound **1** might be derived from its hydroxyl and methoxyl groups in the *ortho* position of on ring A′, B, and B′ (Figure 1) [44]. It has been shown that the number and position of hydroxyl and methoxyl groups contribute to the antioxidant properties of polyphenolic compounds [45,46,47]. Our results agree with previous reports on the remarkable radical scavenging properties of bibenzyl derivative from *Dendrobium* spp. [48,49,50], suggesting that the bisbibenzyl structure is a key determinant of free radical scavenging and protection from oxidative stress. Therefore, the bisbibenzyl structure of compound **1** could be a determinant factor for free radical scavenging and protective activities against oxidative stress on HaCaT cells.

GPx and CAT (enzymatic antioxidants), and GSH (a non-enzymatic antioxidant), are an important role in preventing intracellular organelles against the overproduction of ROS [51]. These antioxidant enzymes can eliminate H_2_O_2_ produced by metabolism or oxidative stress by converting H_2_O_2_ into H_2_O or oxygen, respectively [5,6,7]. Therefore, we then investigated whether the preventive effect of compound **1** is associated with the balance of the antioxidant enzymes on HaCaT keratinocytes. We found that compound **1** could enhance the activities of GPx and CAT and the level of GSH (Figure 4A–C) against H_2_O_2_-induced oxidative stress of HaCaT keratinocytes in a dose-dependent manner. Our result is consistent with the previous study showing that bibenzyl-dihydrophenanthrene from *D. parishii* can improve the activities of antioxidant enzymes (GPx and CAT) [50]. Resveratrol, a well-known stilbenoid, was also reported to increase GPx and GSH activities in HaCaT keratinocytes [52,53]. Compounds with stilbenoid structure have been reported for potent antioxidant activity and antioxidative stress [54,55]. The polyphenolic groups indirectly affect antioxidative stress by improving or inducing signal translocation, which increases antioxidant enzyme expression [53,56,57]. Thus, our data implies that compound **1** prevented HaCaT keratinocytes from oxidative stress by increasing the activities of antioxidant enzymes.

The oxidative stress caused by ROS can lead to the disruption of intracellular redox imbalance and irreversible oxidative modification of macromolecules, leading to the activation of mitochondrial apoptosis programs [58]. Initiation of signaling programmed cell death by ROS leads to stimulation of p53 protein; thereby induces expression of Bax protein and inhibits anti-apoptotic Bcl-2 protein [59]. The activating Bax and inactivating Bcl-2 subsequently disrupt mitochondrial outer membrane permeability, stimulate the release of cytochrome C and other pro-apoptotic factors into the cytosol, and activate the caspase cascade [60]. The major caspase in the apoptosis pathway includes caspase-3 and-9, which act as an effector and an initiator caspase, respectively [61,62,63,64,65]. Our western blot and enzymatic activity assays clearly showed that supplementation with compound **1** inhibits H_2_O_2_-induced-apoptosis through p53/Bax/Bcl2-dependent pathway (Figure 5). Taken together, it is possible that the compound protected HaCaT cells from oxidative stress by either reducing ROS levels or enhancing antioxidant enzyme expressions [66], resulting in the blockade of mitochondria-mediated oxidative stress and apoptosis. The information from this study supports the translational applications of compound **1** in cosmeceutical skin-products.

## 5. Conclusions

**Figure 6:**
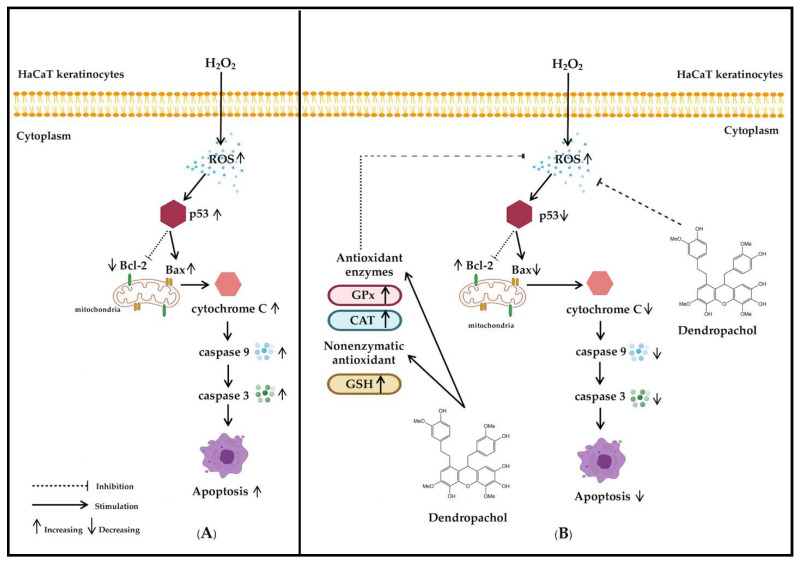
(**A**) Intrinsic pathway of oxidative stress in HaCaT keratinocytes was induced with H_2_O_2_. Enhancement of H_2_O_2_ stimulated ROS production led to the protein’s expression of the p53, Bax, cytochrome C, caspase-9 and caspase-3 due to inhibition anti-apoptotic of Bcl-2. (**B**) The molecular mechanism of compound **1** inhibited H_2_O_2_-induced oxidative stress and increased antioxidant enzymes (GPx, and CAT) and level of non-enzymatic antioxidants activities (GPx) in HaCaT keratinocytes. The intracellular ROS production was decreased from compound **1**, GPx, CAT, and GSH. These lead to the decrease of p53, Bax, cytochrome C, caspase-9 and caspase-3 levels and the increase of the anti-apoptotic of Bcl-2 protein.

In the present study, chromatographic separation of a methanolic extract of *D. pachyglossum* led to the isolation of five compounds (**1–5**), including a new bisbibenzyl named dendropachol (**1**) and four known compounds (**2–5**) comprising 4,5-dihydroxy-2,3-dimethoxy-9,10-dihydrophenanthrene (**2**), gigantol (**3**), moscatilin (**4**) and 4,5,4′-trihydroxy-3,3′-dimethoxybibenzyl (**5**). Compound **1** showed strong free radical scavenging activity, as determined by DPPH assays. Under oxidative stress condition, pre-treatment with compound **1** provides a protective effect against oxidative stress induced by H_2_O_2_ in HaCaT keratinocytes via its ability to scavenge free radicals, improve the activity of antioxidant enzymes (GPx and CAT), and level of non-enzymatic antioxidant (GSH); inhibition of p53-Bax/Bcl2-mediated apoptosis (Figure 6). Compound **1** can, therefore, be considered as potential skincare and rejuvenation agent.

## Figures and Tables

**Table 1:** ^1^H (500 MHz) and ^13^C NMR (125 MHz) spectral data of compound **1** in acetone-*d*_6_.

Position	^1^H	^13^C	HMBC (Correlation with ^1^H)
1	-	137.4	1-OMe
2	-	136.9	4
3	-	141.7	4 *
4	6.21 (s)	109.7	7
5	-	117.9	7 *, 8
6	-	140.0	4, 7
7	4.12 (dd, *J* = 7.0, 5.5 Hz)	39.7	4, 8 *
8	2.67 (m), 2.74 (m)	45.9	7 *, 10, 14
9	-	130.9	7, 8 *, 13
10	6.13 (d, *J* = 2.0 Hz)	114.2	8, 14
11	-	147.5	13, 11-OMe
12	-	145.8	10, 14
13	6.56 (d, *J* = 8.0 Hz)	115.1	-
14	6.22 (dd, *J* = 8.0, 2.0 Hz)	122.9	8, 10
1′	-	147.2	6′ *, 1′-OMe
2′	-	134.0	6′
3′	-	142.4	7
4′	-	119.0	7 *, 8, 6′, 7′
5′	-	129.8	7, 8′
6′	6.66 (s)	108.5	7′
7′	2.76 (m), 2.84 (m)	34.5	6′, 8′ *
8′	2.80 (m)	38.0	7′ *, 10′, 14′
9′	-	134.2	8′ *, 13′
10′	6.77 (d, *J* = 2.0 Hz)	112.9	8′, 14′
11′	-	148.1	13′, 11′-OMe
12′	-	145.6	10′, 14′
13′	6.71 (d, *J* = 8.5 Hz)	115.6	-
14′	6.69 (dd, *J* = 8.5, 2.0 Hz)	121.6	8′, 10′
MeO-1	3.89 (s)	61.1	-
MeO-1′	3.81 (s)	56.7	-
MeO-11	3.56 (s)	55.9	-
MeO-11′	3.76 (s)	56.2	-

* Two-Bond coupling.

## Data Availability

Data is contained within the article.
